# Nanomedicine: The Effective Role of Nanomaterials in Healthcare from Diagnosis to Therapy

**DOI:** 10.3390/pharmaceutics17080987

**Published:** 2025-07-30

**Authors:** Raisa Nazir Ahmed Kazi, Ibrahim W. Hasani, Doaa S. R. Khafaga, Samer Kabba, Mohd Farhan, Mohammad Aatif, Ghazala Muteeb, Yosri A. Fahim

**Affiliations:** 1 Department of Respiratory Therapy, College of Applied Medical Sciences, King Faisal University, Al-Ahsa 31982, Saudi Arabia; rnahmed@kfu.edu.sa; 2 Department of Pharmaceutics, Faculty of Pharmacy, AL-Shamal Private University (S.P.U.), Mari Private University (M.P.U.) and Idlib University, Idlib 5100, Syria; dr.abrahimh@gmail.com; 3 Department of Basic Medical Sciences, Health Sector, Galala University, Suez 43511, Egypt; doaa.rashwan@gu.edu.eg; 4 Department of Pharmaceutical Technology, AL-Shamal Private University (S.P.U.), Idlib 5100, Syria; samerkabba2020@gmail.com; 5 Department of Chemistry, College of Science, King Faisal University, Al-Ahsa 31982, Saudi Arabia; mfarhan@kfu.edu.sa; 6 Department of Public Health, College of Applied Medical Sciences, King Faisal University, Al-Ahsa 31982, Saudi Arabia; maahmad@kfu.edu.sa; 7 Department of Nursing, College of Applied Medical Sciences, King Faisal University, Al-Ahsa 31982, Saudi Arabia

**Keywords:** nanomedicine, healthcare, nanozyme, nanobots, drug delivery system

## Abstract

Nanotechnology is revolutionizing medicine by enabling highly precise diagnostics, targeted therapies, and personalized healthcare solutions. This review explores the multifaceted applications of nanotechnology across medical fields such as oncology and infectious disease control. Engineered nanoparticles (NPs), such as liposomes, polymeric carriers, and carbon-based nanomaterials, enhance drug solubility, protect therapeutic agents from degradation, and enable site-specific delivery, thereby reducing toxicity to healthy tissues. In diagnostics, nanosensors and contrast agents provide ultra-sensitive detection of biomarkers, supporting early diagnosis and real-time monitoring. Nanotechnology also contributes to regenerative medicine, antimicrobial therapies, wearable devices, and theranostics, which integrate treatment and diagnosis into unified systems. Advanced innovations such as nanobots and smart nanosystems further extend these capabilities, enabling responsive drug delivery and minimally invasive interventions. Despite its immense potential, nanomedicine faces challenges, including biocompatibility, environmental safety, manufacturing scalability, and regulatory oversight. Addressing these issues is essential for clinical translation and public acceptance. In summary, nanotechnology offers transformative tools that are reshaping medical diagnostics, therapeutics, and disease prevention. Through continued research and interdisciplinary collaboration, it holds the potential to significantly enhance treatment outcomes, reduce healthcare costs, and usher in a new era of precise and personalized medicine.

## 1. Introduction

Nanotechnology encompasses a broad interdisciplinary field of science and engineering focused on understanding and manipulating matter at the atomic and molecular scale [1]. At its core, it involves the design, development, and deployment of materials and devices with dimensions measured in nanometers [2]. The theoretical foundation of this field was first introduced by physicist Richard Feynman in 1959, who envisioned manipulating individual atoms and molecules to create new materials and technologies [3]. Since then, nanotechnology has rapidly evolved and is now considered one of the most transformative scientific advancements of the 21st century, especially in the field of medicine. Over the past few decades, the potential of nanotechnology to revolutionize healthcare has garnered increasing attention, resulting in significant investment and research globally [4]. Governments and institutions have ramped up funding initiatives, recognizing nanotechnology as a catalyst for innovation, economic development, and societal well-being. The profound impact of nanotechnology extends across industries, promising to reshape how we approach treatment, diagnostics, and disease prevention [5].

With nanotechnology, a new frontier in medical science has emerged, one that allows for precision interventions at the cellular and molecular level [6]. Engineered nanodevices and nanoscale systems are now being explored for their ability to monitor, repair, and protect biological systems in unprecedented ways. These innovations aim to reshape conventional medicine, advancing human health and longevity [7].

The influence of nanotechnology on the life sciences is profound. It enables breakthroughs across various branches of healthcare, from early disease detection to the creation of vaccines and minimally invasive surgical tools [8]. Its ability to manipulate matter at the molecular scale has ushered in a new era of personalized medicine, where therapies can be tailored to an individual’s genetic and cellular profile [9]. The application of nanotechnology in healthcare spans a wide spectrum. In particular, nano-based therapeutics are showing promise in enhancing drug bioavailability, minimizing side effects, and improving the targeted delivery of medications [10]. For instance, the blood–brain barrier, which acts as a selective gateway to the brain, has traditionally posed a challenge to drug delivery. However, specially engineered nanoparticles are now capable of crossing this barrier, offering new treatment strategies for neurological conditions [11]. Conventional treatments for conditions such as vascular thrombosis often suffer from limitations, including short circulation times and adverse effects. Nanotechnology-based delivery systems such as polymeric nanoparticles and liposomes can encapsulate drugs to increase stability and therapeutic efficacy. These carriers are biocompatible and biodegradable, making them ideal candidates for advanced drug delivery applications [12].

Nanomedicine represents a transformative frontier in the medical sciences, harnessing the precision and power of nanoscale technologies to tackle complex diseases such as cancer, cardiovascular disorders, and a host of other serious health conditions [13]. With recent progress in nanotechnology, clinicians and researchers are now able to target biological processes within the human body at an unprecedented molecular level, utilizing materials such as biocompatible nanoparticles and nanoscale robotic devices [14]. The integration of nanotechnology has significantly broadened the scope and commercial potential of nanomedicine, offering tools like nanosensors and nanomachines capable of assessing biochemical activities within organs and accessing diseased tissues with remarkable precision [15].

One of the core strengths of nanomedical systems lies in their ability to shield therapeutic agents from degradation, thus prolonging their bioactivity and enhancing water solubility. This protection ensures that drugs reach their target intact and remain effective within the body’s harsh internal environment [16]. In diagnostics, nanoparticles serve as powerful tools for detecting biomarkers, including those associated with tumors and genetic conditions, enabling earlier and more accurate diagnosis. Applications of nanomedicine extend across a wide spectrum from traditional chemotherapy and biologic agents to next-generation immunotherapies [17]. Nanostructured carriers can be engineered to recognize disease-specific molecular signals, facilitating highly selective targeting that minimizes harm to healthy cells. This has proven particularly impactful in oncology, where nanocarriers enhance drug delivery efficiency while mitigating adverse side effects [18].

Across the vast ecosystem of nanotechnology, nearly every sector, from pharmaceuticals to diagnostic machinery, is experiencing rapid innovation. Yet, with this growth comes a rising concern over the societal and economic implications of widespread nanotech use [4]. The long-term health and environmental impacts remain subjects of ongoing research and public debate, often outpacing public awareness and regulatory preparedness [19].

In modern medicine, nanotechnology is utilized for targeted drug delivery, enabling therapeutic agents to be directed precisely to diseased tissues or cells. This precision not only boosts treatment outcomes but also reduces damage to surrounding healthy tissues [20]. Nanoprobes, smart nanoelectronic sensors, and multifunctional nanoparticles represent cutting-edge advancements in this domain, capable of integrating diagnostics with therapy in a concept known as “theranostics” [21]. Remarkable progress has been made in refining intracellular drug transport and controlled gene release using nanoparticles. Such advancements allow clinicians to fine-tune dosages and enhance treatment effectiveness [22]. Moreover, nanotechnology is playing a pivotal role in regenerative medicine, with innovations such as magnetic nanoparticles aiding neural regeneration and enzyme-responsive nanosystems designed for brain tumor therapy [23].

Nanorobotics, a cutting-edge application within nanomedicine, is especially promising. These microscopic machines are being developed to perform tasks such as real-time disease monitoring, optimizing drug distribution, and supporting precision medicine [24]. Their ability to self-assemble and maintain complex systems inside the body may lead to revolutionary improvements in therapeutic outcomes. Additionally, the increasing reliance on nanorobotics is fueling expansion in the medical technology market, signaling a shift toward more efficient, responsive, and personalized healthcare solutions [25]. Nanozymes, an emerging class of nanomaterials, combine the catalytic activity of enzymes with the distinct physicochemical features of nanoscale molecules [26]. This study seeks to give a complete overview of nanomedicine’s involvement across the illness management continuum, from early detection to tailored treatment. It focuses on current advancements in nanoscale diagnostic tools, drug delivery systems, and therapeutic nanoplatforms, as well as their clinical promise, limitations, and future prospects. By connecting nanotechnology’s diagnostic and therapeutic uses, the paper underlines nanomedicine’s integrative potential in improving patient outcomes and promoting personalized medicine.

## 2. Methodology

The methodology of this review involved a comprehensive and systematic search of the scientific literature to gather current information on the diagnostic and therapeutic applications of nanotechnology in medicine and healthcare. A targeted search was conducted across major databases, including PubMed, Scopus, Web of Science, and Google Scholar. Keywords used in various combinations included “nanotechnology,” “nanomedicine,” “nanoparticles,” “diagnostics,” “drug delivery,” “therapeutics,” and “biomedical applications.” Only English-language articles were considered, and studies were selected based on their relevance to medical diagnostics and therapeutics, with a preference for original research, clinical trials, and high-impact reviews. Exclusion criteria included non-medical applications of nanotechnology, editorials, and articles lacking experimental or clinical data. After an initial screening of titles and abstracts, a full-text evaluation was conducted, and data were extracted regarding the types of nanomaterials, their mechanisms of action, clinical targets, and reported outcomes. Studies were grouped into diagnostic and therapeutic categories and analyzed for innovation, translational value, and scientific quality to provide a balanced and insightful synthesis of the current state of nanotechnology in healthcare.

## 3. Nanomedicine

**Figure 1:**
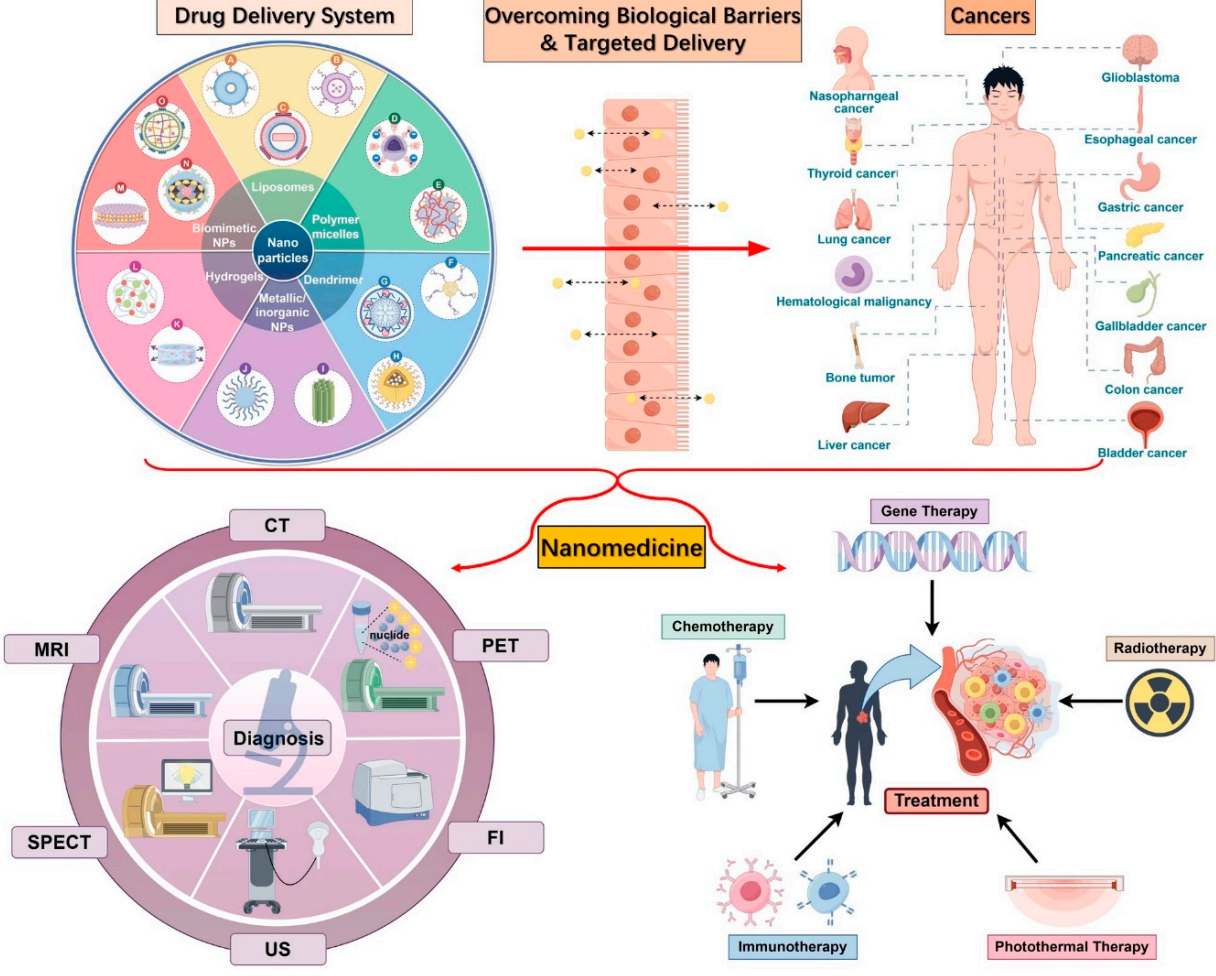
Nanotechnology applications in the medical field. CT: computed tomography, MRI: magnetic resonance imaging, PET: positron emission tomography, SPECT: single-photon emission tomography, US: ultrasound, and FI: fluorescence imaging [37].

The integration of nanotechnology into the medical domain has triggered a paradigm shift in how diseases are diagnosed, monitored, and treated, as illustrated in Figure 1. As the capabilities of nanomedicine continue to expand, they are redefining traditional healthcare models, offering innovative solutions with far-reaching impacts on patient outcomes [27]. The ongoing development of nanoscale drug delivery systems and diagnostic tools demonstrates the urgent need to further explore and implement these technologies in clinical settings [28]. Nanotechnology has enabled the evolution of drug therapy by introducing more precise and effective treatments. Areas such as oncology, cardiology, nephrology, and genetic medicine have witnessed transformative progress due to the application of nanoscale materials and engineered systems [29]. For example, gene therapy has leveraged nanoparticles to improve delivery efficiency, often utilizing viral vectors engineered at the nanoscale to transport therapeutic agents directly to targeted cells [30].

Smart devices such as ingestible sensors, nanobots, and responsive drug delivery systems are now capable of monitoring patient health in real time. These devices can provide critical feedback to healthcare professionals, ensuring the accurate administration of medications and enhancing the overall effectiveness of therapeutic regimens. The word “smart” refers to the capability of these devices to gather information intelligently and instantly. Polymeric nanoparticles designed for inflammatory bowel disease (IBD) are primarily smart in nature. They can react to various stimuli, including reactive oxygen species, pH levels, specific ligands, magnetic fields, and the gut microbiome, enabling controlled drug release. By encapsulating the medication, they can safeguard it, minimize side effects, and specifically target certain cells. Smart nanoparticles have surfaced as a potential replacement for traditional nanoparticles in cancer treatment. In contrast to conventional nanoparticles, they can be activated by particular stimuli and deliver drugs accurately to targeted sites [31,32,33]. By replacing conventional diagnostic procedures with nanoparticle-enabled assays, medical practitioners can achieve higher accuracy at reduced costs. Nanoparticles are increasingly used as contrast agents or imaging enhancers, especially in identifying cancer biomarkers and tracking cellular changes in real-time. These particles can be fine-tuned to detect specific genetic mutations and cellular behaviors associated with tumor development [34].

Medical devices now often feature nanoscale surface coatings made from compounds such as titanium dioxide, silicon dioxide, zinc oxide, silver, and iron oxide. These coatings improve device performance by enhancing durability, biocompatibility, and resistance to microbial contamination [35]. Advancements in material science, particularly in nanosensors and nanostructured energy systems, are driving significant improvements in the design and performance of medical devices. These innovations underscore the growing importance of nanotechnology in medical engineering. Through collaborative efforts, scientists and engineers are developing next-generation medical technologies that integrate these cutting-edge materials, leading to smarter diagnostic tools, safer implantable devices, and more effective therapeutic systems [36].

## 4. Various Types of Nanoparticles That Are Used in Medical Applications

**Figure 2:**
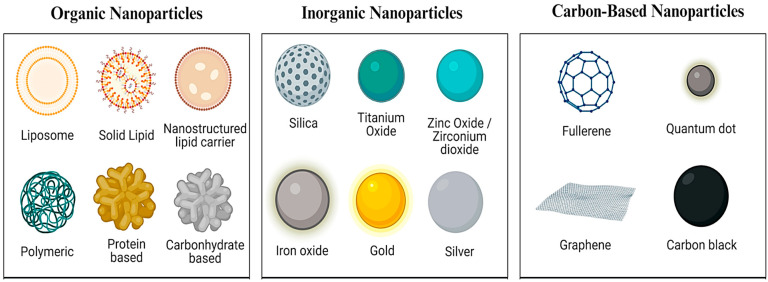
Different types of nanoparticles used in the medical field [45].

Nanoparticles are distinguished by their high surface-area-to-volume ratio, a characteristic that allows for superior drug loading capacity and enhanced circulation through the bloodstream. Their nanoscale size grants them unique physical and chemical properties, including tailored optical, magnetic, mechanical, and catalytic behaviors, making them ideal candidates for various biomedical applications [38]. The following are some of the significant technological benefits of nanoparticles as drug carriers: high stability (i.e., long shelf life); high carrier capacity (i.e., many drug molecules can be incorporated in the particle matrix); the feasibility of incorporating both hydrophilic and hydrophobic substances; and the feasibility of multiple routes of administration, including oral and inhalation. These carriers can also be developed to permit regulated (long-term) medication release from the matrix [39]. Nanoparticles can be engineered to respond to specific stimuli such as light, temperature, or magnetic fields. For instance, certain metal-based particles absorb light and convert it into heat, a feature that can be used to selectively destroy cancer cells, a process known as photothermal therapy [40]. Based on their chemical composition, as illustrated in Figure 2, nanoparticles used in medicine are generally categorized into three primary groups: organic, inorganic, and carbon-based nanomaterials.

- Organic nanoparticles are composed of biologically derived substances such as proteins, lipids, carbohydrates, and biodegradable polymers [41]. These particles are often engineered to be smaller than 100 nanometers and are valued for their compatibility with human tissues, making them effective carriers for drug molecules and genetic material [42].
- Inorganic nanoparticles encompass materials such as metal salts, metal oxides, and pure elemental metals. These particles are known for their chemical stability, water solubility, and biocompatibility. In comparison to organic NPs, they offer enhanced control over particle behavior and are less prone to degradation [43].
- Carbon-based nanoparticles include structures like fullerenes, graphene derivatives, and carbon nanotubes. These materials are prized for their remarkable thermal, electrical, and mechanical strength, along with their adaptability for biomedical functions such as targeted drug delivery, tissue scaffolding, and biosensing [44].

## 5. Key Attributes and Functional Characteristics of Nanotechnology in Medicine

Nanotechnology brings forth a suite of distinctive attributes that are transforming healthcare, particularly in areas such as wound care, antibacterial therapies, targeted treatment delivery, and diagnostic innovation [46]. These nanotechnological traits make medical interventions more accurate, less invasive, and more effective across both preventive and reactive care models. In addition to therapeutic functions, wearable nanosensors are contributing significantly to chronic care, especially for elderly populations in remote regions. These devices can continuously transmit biometric data to healthcare providers, ensuring prompt interventions.

### 5.1. Wound Treatment and Infection Control

Significant progress has been achieved in tissue regeneration and wound healing through the use of nanotechnology. Engineered nanomaterials can now assist the body in repairing damaged tissues by providing scaffolds that closely resemble the structure of natural cells [47]. This is particularly significant in reconstructive surgeries where traditional grafts or transplants may not suffice. Nanoparticles can be functionalized with specific ligands and imaging agents to selectively bind to diseased tissue, allowing for accurate identification of injuries and optimized surgical precision, which assists medical professionals in differentiating between healthy and affected regions, reducing the risk of further damage [48]. At the nanoscale, materials exhibit properties that differ drastically from their bulk counterparts, such as enhanced reactivity or permeability. These characteristics are leveraged to develop treatments that can penetrate biological barriers and interact directly with intracellular components, such as DNA or proteins, improving the effectiveness of therapeutics. Additionally, nanogenerators and polymer-based nanomaterials are being explored to stimulate wound healing processes more rapidly and with greater success [49]. With rising antibiotic resistance, nanotechnology offers alternative solutions to traditional antimicrobial strategies [50]. Materials like silver nanoparticles exhibit high antibacterial activity and can be incorporated into coatings, dressings, and devices to prevent infections [51]. These innovations help reduce dependency on conventional antibiotics, lowering the risk of resistance development. Additionally, nanomedicine introduces new methods for managing chronic illnesses more effectively, particularly in resource-limited settings [52].

**Figure 3:**
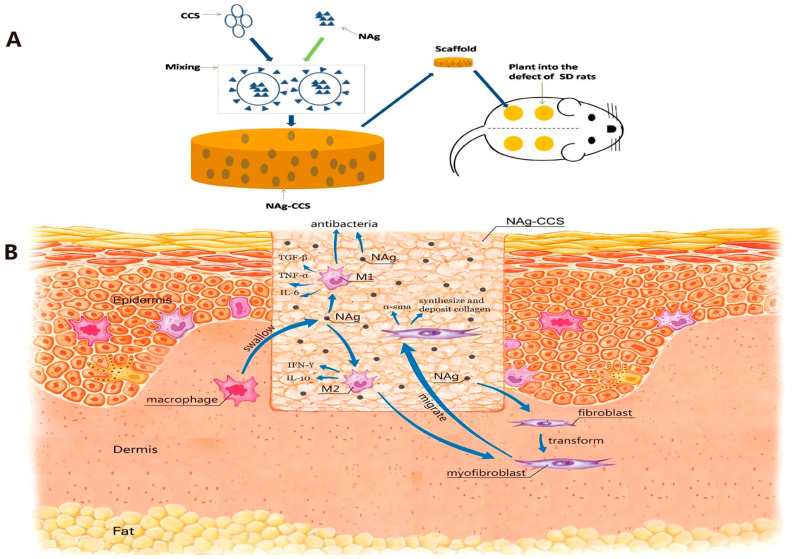
(**A**) A graphical representation of the NAg-CCS transplanted into the defects of male Sprague–Dawley (SD) rats. (**B**) Possible mechanisms for NAg-accelerated cutaneous wound healing [53].

In previous research, a new NAg-CCS was created by combining silver nanoparticles (NAg) with collagen–chitosan scaffold (CCS), as illustrated in Figure 3A, a dermal scaffold with high mechanical strength, flexibility, tear resistance, and biocompatibility. In vitro and in vivo investigations were carried out to assess the antibacterial properties of NAg-CCS and its involvement in wound healing. Figure 3B depicts the proposed modes of action for NAg [53].

### 5.2. Antimicrobial Treatments

**Figure 4:**
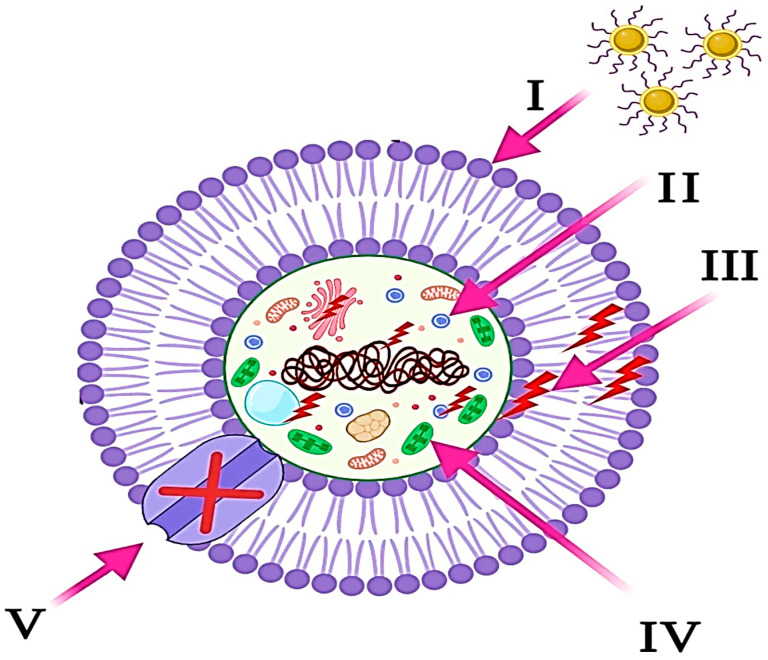
The diagram illustrates the five primary mechanisms by which NPs exhibit their antibacterial properties. (I) The NPs stick to and attach to the surface of the microbial cell, which in turn causes damage to the cell membrane and changes in its transport activity. (II) NPs infiltrate microbial cells and engage with intracellular biomolecules, thereby influencing the corresponding cellular machinery. (III) The presence of NPs induces the generation and amplification of ROS, resulting in cellular damage. (IV) The NPs manipulate the cellular signal pathway and induce cell death. (V) NPs effectively inhibit the movement of ions into and out of microbial cells [61].

The antibacterial potential of nanomaterials is receiving growing attention as a promising strategy to combat the escalating challenge of antimicrobial resistance. Antimicrobial resistance mechanisms include active efflux pumps, restricted permeability of bacterial membranes, enzymatic degradation of antimicrobial agents, the formation of protective biofilms, altered antimicrobial targets, and the protection of bacterial sites of action from antimicrobials. Nanotechnology enables the development of highly effective antimicrobial agents and surfaces [54], as illustrated in Figure 4. For instance, nanoparticles composed of gold, silver, and quantum dots—when activated by infrared light—can be employed to sterilize equipment or inhibit bacterial proliferation on medical instruments and implants [55]. Nanomaterials are also incorporated into wearable diagnostic platforms and vaccine delivery systems to enhance immune responses and prevent infections. These innovations not only help in the detection of pathogens but also in preventing their spread by disrupting microbial biofilms and membrane structures [56]. At the core of these capabilities is the manipulation of materials at the molecular scale, which allows for the creation of uniquely responsive therapeutic agents that outperform conventional antibiotics in both effectiveness and resistance management [57], as shown in Table 1. Beyond traditional nanoparticles, a broader array of nanomaterials is being explored for their antimicrobial efficacy. Hydrogels, for instance, possess a porous structure suitable for loading antibiotic drugs [58]; nanofibrous scaffolds have a number of inherent features that make them uniquely beneficial for antibacterial applications [59]; and carbon-based nanomaterials, such as carbon nanotubes, have recently gained a lot of attention for their antibacterial properties [60].

### 5.3. Minimizing Cytotoxic Effects on Healthy Cells

All medications have side effects, although their magnitude and severity range from mild to severe. The majority of adverse effects are expected and are listed. However, the main issue is that some of the treatments’ side effects are unknown or have not been observed, and the actual concern here is whether they will have a severe negative influence on the people who use them. The most crucial elements that may enhance the severity of the side effects are the kind of drug and the type of people using them [68]. A significant advantage of nanotechnology in cancer treatment is its capacity to precisely deliver drugs to malignant cells while sparing surrounding healthy tissues [69]. By engineering nanoparticles to recognize specific tumor markers, treatments can be focused on diseased areas, improving therapeutic outcomes and minimizing adverse side effects commonly seen in chemotherapy. For example, nanotubes conjugated with tumor-specific antibodies can be directed to cancer cells. Upon exposure to a laser, these nanotubes absorb the energy and generate localized heat, destroying the tumor without harming nearby healthy tissue [70]. This method of combining photothermal therapy with nanotechnology exemplifies a leap forward in selective treatment delivery. Furthermore, hybrid systems utilizing both biological and synthetic materials are being explored to improve compatibility, reduce immune responses, and enhance treatment effectiveness through biomimetic strategies [71]. The liposomal drug formulations reduced toxicity to healthy cells [72]. Dendrimers have been shown to increase biocompatibility in biological systems, including in vitro and in vivo cytotoxicity, immunogenicity, and bio-stability [73]. Gold nanoparticles are attractive prospects for numerous cancer therapy and diagnostic applications, including medical imaging, drug transport, and light-activated treatments, due to their comparatively low toxicity and unique, adjustable optical characteristics [74].

### 5.4. Nanomedicine Diagnostic Technique

The diagnostic capabilities empowered by nanotechnology are rapidly reshaping how diseases are identified and monitored. Techniques employing gold nanorods, carbon nanotubes, and other nanosensors now allow for ultra-sensitive, cost-effective, and rapid detection of diseases at their earliest stages [75]. These innovations mimic natural biological behavior more closely than traditional prosthetics, supporting cellular growth and functionality in a more seamless way. The integration of nanodiagnostic platforms with therapeutic systems paves the way for advanced theranostics, a synergistic approach that combines diagnosis and therapy. This enables real-time monitoring of treatment responses, allowing dynamic adjustments to therapeutic regimens to optimize efficacy and improve clinical outcomes [76].

Recent years have witnessed a leap in diagnostic imaging technologies such as MRI and CT scans. Yet, nanotechnology is pushing beyond the current limits of conventional tools by offering ultra-sensitive diagnostic techniques for both in vivo and in vitro applications [77]. Nanoscale diagnostic agents can access and analyze biomarkers at cellular and even subcellular levels, potentially enabling physicians to identify disease long before symptoms manifest. This precision will be key in early-stage disease intervention and personalized treatment planning [78].

Nanomedicine is revolutionizing disease detection by enabling highly sensitive, specific, and early identification of pathological changes at the molecular level. Smart nanoparticles can be functionalized with targeting ligands to bind selectively to disease biomarkers, allowing for real-time imaging and diagnosis using advanced modalities such as MRI, PET, fluorescence, and photoacoustic imaging [79,80,81]. Quantum dots and other fluorescent nanomaterials enhance molecular imaging precision, while nano-based biosensors using materials like gold nanoparticles, carbon nanotubes, or graphene offer rapid, point-of-care detection of biomarkers in bodily fluids with minimal invasiveness [82,83]. Moreover, nanotechnology enables integration with implantable devices to monitor biochemical changes such as pH, glucose, or inflammatory signals, enhancing tissue compatibility and early complication detection [84]. Liquid biopsy techniques, powered by nanoparticles, allow dynamic monitoring of circulating tumor cells, exosomes, and tumor DNA, supporting personalized treatment strategies [85]. The unique surface properties of nanomaterials also support multiplexed diagnostics and smart sensor networks, which, when paired with AI-based analytics, pave the way for predictive and preventive healthcare. Overall, nanodiagnostics is ushering in a new era of real-time, precise, and personalized disease monitoring and detection [86].

Nanosensors are being developed to detect vital physiological parameters such as oxygen and carbon dioxide levels, as well as harmful substances within the body [87]. These devices can be used to assess conditions like hypoxia, detect malignancies in the digestive tract, and help tailor dietary recommendations based on food intolerances. New techniques such as NanoFlares a nanotechnology-based method to detect cancer cells by targeting genetic markers, are demonstrating the power of nanoscale tools in improving diagnostic accuracy while minimizing side effects [88].

## 6. Emerging Frontiers of Nanotechnology in Modern Medicine

Nanotechnology is redefining medical science through innovations in diagnostics, precision treatment, and advanced therapies [89]. Compact, portable systems now allow real-time analysis of minimal biological samples, drastically reducing the time required for diagnostics. As biosensor technologies evolve, their sensitivity and capability to process larger volumes of data will continue to enhance early disease detection [90]. Formulations using iron oxide particles and functional polymers have significantly improved imaging quality, even at lower dosages, thus reducing patient exposure to contrast agents while increasing the precision of diagnosis, especially in the identification of genetic disorders and malignancies [91]. Although the potential of nanomedicine is enormous, concerns regarding safety, ethics, and privacy remain critical. Advanced systems like regenerative immune biosensors are being designed for continuous monitoring with greater statistical robustness. In oncology, for example, nanomedicine is still emerging, and more clinical validation is necessary to fully understand its long-term impact and reliability [92].

### 6.1. Tissue Engineering and Cell Treatment

In tissue engineering, nanotechnology is employed to replicate the microenvironment of natural tissues through engineered scaffolds that support cellular growth and differentiation [93]. These nanostructures serve as platforms for cell adhesion and regeneration, enabling the formation of synthetic tissues that closely resemble their natural counterparts. Biocompatible nanomaterials not only promote healthy tissue development but also facilitate control over cellular repair and regeneration processes, thereby playing a critical role in organ restoration and wound healing therapies [47,94]. Nanofibers are fibers with sizes in the nanometric range. Biomaterial-derived nanofibers have the qualities of both nanofibers and renewable polymers, including a high surface-to-volume ratio, excellent cell adhesion, proliferation, and differentiation, and extracellular matrix movement capabilities. These are widely employed in tissue engineering and biomedical applications [95]. In recent years, significant progress has been achieved in the production of hydrogels incorporating nanocomposites. These hydrogels are easy to manufacture, have good mechanical characteristics, and can boost the stimulation response via the synergistic action of nanoparticles; hence, they have gained great attention in the tissue engineering field [96].

### 6.2. Targeted Drug Delivery

Advanced drug delivery mechanisms enabled by nanotechnology involve nanoscale carriers embedded with active pharmaceutical ingredients [97,98]. These carriers are tailored to reach specific tissues or organs, thereby maximizing drug impact and minimizing unwanted effects [99,100]. Metallic, organic, inorganic, and polymeric nanostructures, such as dendrimers, micelles, and liposomes, are commonly used in the creation of targeted drug delivery systems. These nanoparticles are specifically used to tag medicines with low solubility and absorption. However, the efficiency of these nanostructures as drug delivery vehicles varies with their size, shape, and other underlying biophysical/chemical properties [10]. Polymeric nanoparticles, such as PLGA (poly lactic-co-glycolic acid) systems, allow for controlled and sustained drug release, reducing the frequency of administration and improving patient adherence to treatment regimens [101].

There are two types of targeted medication delivery: active and passive. While active targeted drug delivery is dependent on ligand interactions with receptors, the passive method depends on the increased permeability and retention effect (EPR effect). Active targeted medication delivery is based on the interaction of ligands with receptors [69]. The medication nanoparticles include particular ligands capable of recognizing certain receptors, which are more concentrated or significantly higher in abnormal cells than in healthy cells. This guarantees that the drug-NP concentrates just in the abnormal region. Nanomaterials are also being used to detect and isolate circulating tumor cells, offering hope in early cancer detection and metastasis control [102]. Cancer cells, for example, have receptors that vary from those found in normal tissues. Overexpression of receptors on cancer cells allows them to satisfy their nutritional demands and continue their hypermetabolism. Passive targeted delivery uses leaky vasculature and poor lymphatic drainage to provide selective and increased distribution in abnormal cells. Diseased cells, such as tumors, develop leaky blood arteries, making them more permeable than normal cells. Due to the size of the medications, their leaky nature may prevent them from accumulating. Second, the lack of normal lymphatic outflow in tumors adds to NP retention. As a result, coupling medicines to NPs can enhance their penetration and retention in cancer cells. This eventually enhances medication bioavailability and extends the duration of action of the medicine at target areas, resulting in fewer adverse effects [103]. Anticancer therapy fails because cancer cells are resistant to drugs. Increased drug efflux, evasion of drug-induced apoptosis, and activation of DNA repair mechanisms can all contribute to chemotherapy resistance. Several initiatives to combat MDR in cancer have failed in recent years. Understanding MDR and cellular reprogramming might help us overcome cancer drug resistance and enhance cancer treatment. Cancer multidrug resistance can be treated using new anticancer drug candidates, molecular targets, and breakthrough drug delivery systems (DDSs) [104], as shown in Table 2.

## 7. Advanced Nanotechnology Applications in Medicine and Therapy

### 7.1. Nanobots

**Figure 5:**
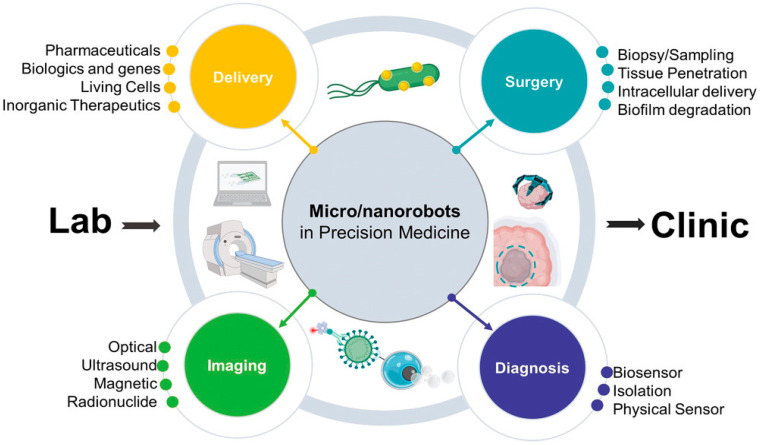
Current trends of micro/nanorobotics in precision medicine [116].

Among the most transformative innovations in nanomedicine is the development of nanorobotics, microscopic machines capable of performing intricate tasks at the cellular and molecular levels [112], as illustrated in Figure 5. These nanobots are envisioned as programmable agents that can repair or replace malfunctioning cell components, correct genetic mutations, or even substitute segments of DNA to eliminate hereditary diseases [113]. In therapeutic contexts, nanobots may be deployed to clear arterial blockages or assist in organ regeneration. Unlike passive nanocarriers, nanobots can actively navigate the body, guided by AI algorithms that enable them to communicate, coordinate actions, and respond adaptively to their biological environment [114]. Each unit could operate independently or in swarms, exchanging information and dynamically altering their structure, cargo, or surface properties to improve their effectiveness in detecting, diagnosing, or targeting disease sites [115].

### 7.2. Nanozyme

**Figure 6:**
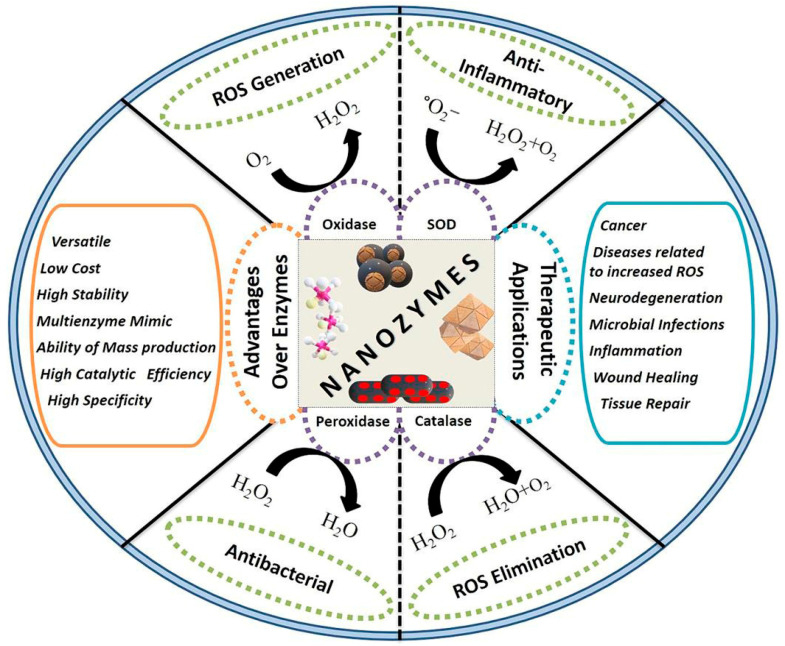
Medical applications of nanozymes [120].

Natural enzymes are well-known for their catalytic activity against certain substrates; yet, they require precise conditions to be stable under severe conditions such as high temperatures. To address this issue, scientists have developed immobilization methods based on nano- and microscale materials. The combination of these materials in healthcare equipment mimics the properties of main biological environments. These nanomaterials can be enhanced using a variety of components, including artificial polymers, carbon-based nanoparticles, metal–organic frameworks, nanosized protein-based nano-delivery systems, lipid-based nanomaterials, and polysaccharide-based nanoparticles. As a result, nanozymes offer the advantages of natural enzymes in addition to better stability, simplicity of storage, and easy production [98]. In recent years, nanozyme research has led to several advances in sensing, antibacterial activity, cancer therapy, antioxidant activity, and environmental treatment. Nanoparticles having intrinsic catalytic activity offer a wide range of biological uses, including detection, diagnostics, treatment, and therapy. Hydrogen peroxidase, metal ions, and other variables influence their activity. Many nanomaterials mimic enzyme-like action, according to nanozyme research, including oxidase (OXD), glucose oxidase (GOD), peroxidase (POD), catalase (CAT), superoxide dismutase (SOD), and glutathione peroxidase (GPx). Nanozyme technology has improved to the point where it can be utilized to generate sophisticated variants in a variety of fields [117], as illustrated in Figure 6. One example is magneto-gold nanozymes (AuNC@Fe_3_O_4_), which display photothermal effects and peroxidase-like activity, enhancing ROS production and causing death in HCC cells by a synergistic mechanism combining photothermal and nano-catalytic treatment [118]. Furthermore, cobalt nanozymes inside ferritin nanocages show potential in prognostic diagnosis by distinguishing HCC tissues from normal tissues with significant sensitivity and specificity [119].

### 7.3. Enhancing Radiation Therapy

Radiation treatment is being refined through the use of nanoparticles engineered to selectively accumulate in tumor tissues. These specialized particles can intensify the effects of radiation precisely at the cancer site, sparing surrounding healthy cells and reducing the harmful side effects commonly associated with conventional therapies [121]. One of the primary goals of integrating classical radiation therapy with nanotechnology is to enhance the difference in effects between cancerous and healthy cells, ensuring that only tumors are impacted by radiation treatment. Researchers are concentrating on specifically targeting cancer cells during radiation application and making radioresistant cancer cells more susceptible to standard radiation doses. Various nanoparticles can contribute to augmenting the sensitivity of cells to radiation by interacting directly with ionizing radiation. These nanoparticles are typically composed of elements that have high atomic mass and atomic number, such as gold, silver, titanium, and platinum. Nanoparticles serve not only as radiosensitizers but also as radioprotective agents to lessen the detrimental effects of radiation on healthy tissues. For instance, nanoparticles that incorporate antioxidants or free radical scavengers can shield normal cells from damage caused by radiation [122].

## 8. Future and Emerging Prospects

The evolving field of nanomedicine is poised to play a foundational role in the next generation of personalized healthcare, offering capabilities that extend from early disease forecasting to real-time health monitoring [123]. As research deepens, nanoscale materials are becoming integral to the design of ultra-sensitive diagnostic tools and molecular-level biomarkers that can detect multiple disease states at the earliest stages with exceptional accuracy and specificity. One of nanomedicine’s key strengths lies in its ability to deliver targeted interventions [124]. Following an accurate diagnosis, nanoparticle-based systems can selectively attack diseased cells while sparing healthy tissues, minimizing collateral damage and side effects. Yet, the future holds even greater promise, particularly in enhancing drug payload capacity, improving release mechanisms, and refining the therapeutic capabilities of metallic nanoparticles [125]. However, as with any disruptive medical technology, the rapid advancement of nanomedicine must be met with robust safety frameworks. Each therapeutic application must undergo comprehensive toxicological assessments and multilayered clinical evaluations before widespread clinical adoption. Regulatory rigor will be essential to balance innovation with safety and ensure responsible integration into mainstream medicine. Looking ahead, nanotechnology may eventually replace reliance on external diagnostic tools and predictive models by directly identifying physiological anomalies at the cellular level [126]. One unique potential application may emerge in sports science, where nanodevices could monitor muscle efficiency, track blood flow, and identify areas of high lactic acid buildup [127]. This insight would allow athletes to fine-tune training regimens, enhance underperforming muscle groups, and optimize physical performance more scientifically. Ultimately, the continued evolution of nanomedicine promises to transform how we prevent, diagnose, and treat disease, ushering in a new era of highly personalized, precise, and proactive medical care.

## 9. Challenges and Limitations

Despite its promising potential, nanomedicine faces several technical, economic, and safety-related hurdles that must be addressed before widespread clinical integration can occur. One of the most pressing issues lies in ensuring consistency in the production of nanomaterials. Achieving batch-to-batch reproducibility, maintaining stringent quality standards, and scaling up manufacturing processes without compromising precision remain formidable tasks for researchers and industry alike. Moreover, the high cost of developing nanomedical products continues to be a barrier, especially in the face of limited clarity about their long-term biological and ecological effects. Uncertainties regarding how nanoparticles interact with the environment and human physiology have raised red flags among regulatory bodies and investors. The pharmaceutical industry, in particular, has been hesitant to make significant financial commitments due to these unresolved risks and the slow pace of regulatory approvals. From a biological standpoint, the use of nanoparticles inside the body introduces a range of complications. These microscopic entities, due to their size and surface properties, can evade immune surveillance, bypass physiological defense systems, and accumulate in specific organs. In some cases, their presence may lead to unintended tissue deposition or toxicity that the body struggles to eliminate. In addition, the generation of waste materials and unwanted by-products during nano-engineering processes introduces further complexity, posing both environmental and biomedical risks that have yet to be fully understood or mitigated. Overall, while nanomedicine offers groundbreaking opportunities, its path to clinical and commercial maturity is still encumbered by scientific uncertainties, high development costs, and a cautious investment climate. Overcoming these limitations will require coordinated efforts in research, policy, and industry to ensure that innovation proceeds safely and sustainably. Despite growing interest in nanomedicine, regulatory monitoring remains one of the most important roadblocks to clinical use. Nanomaterials provide distinct problems because of their dynamic physicochemical features, complicated interactions with biological systems, and unexpected long-term behavior. These qualities make it difficult to analyze their safety, effectiveness, and quality under established pharmaceutical regulatory systems. Regulatory organizations such as the United States Food and Drug Administration (FDA) and the European Medicines Agency (EMA) have identified these problems and are working to establish rules unique to nanotechnology-based goods. The FDA, for example, has issued several guideline documents on nanomaterial characterization, bioequivalence, and risk assessment. Similarly, the EMA has issued reflection papers to aid in the evaluation of nanomedicines and nanosimilars within the EU regulatory framework. However, a globally consistent approach for assessing nanotherapeutics is absent. The main issues include identifying key quality characteristics (CQAs), developing validated models for toxicity assessment, and forecasting nanoparticle biodistribution and clearance. Furthermore, the regulatory classification of nanomedicines—whether as pharmaceuticals, devices, or combination products—may differ among areas, complicating approval procedures. Addressing these gaps in regulation is critical for promoting innovation while maintaining patient safety. Collaboration among regulatory bodies, academic institutions, and industry stakeholders is required to produce consistent, science-based rules that may speed up the safe and successful deployment of nanomedicine globally.

## 10. Conclusions

Nanotechnology is rapidly transforming modern healthcare by shifting the focus from reactive treatment to proactive health management. One of its core strengths lies in enabling precise therapeutic delivery, significantly lowering the risk of unintended side effects while improving overall treatment outcomes. Its precision-driven approach has shown immense promise in the management of complex diseases such as cancer, where accurate targeting is crucial. Among the most innovative advancements is the integration of nanorobotics within medicine, facilitating a wide spectrum of applications. These range from targeted drug transport and smart diagnostic systems to the development of next-generation vaccines and antimicrobial solutions. Additionally, nanotechnology is contributing to the evolution of wearable health monitoring devices and advanced imaging platforms. The synergy between conventional pharmaceutical therapies and nanotechnology is paving the way for a new era of combination treatments that can cross physiological barriers, such as the blood–brain barrier, and act directly on disease sites with heightened specificity. As a result, even long-standing drug classes are being revitalized through nanoscale delivery systems that enhance efficacy and reduce systemic toxicity. Ongoing advancements are also laying the groundwork for personalized medical strategies, where medications and diagnostic tools can be custom-designed based on an individual’s unique biological profile. Through the controlled use of engineered nanomaterials such as manganese-citrate complexes, researchers are creating highly efficient drug carriers and diagnostic modalities that operate at the molecular level. In conclusion, nanomedicine represents a transformative force in healthcare innovation. Its capacity to redefine disease detection, intervention, and prevention holds significant promise for improving global health outcomes and shaping the future of medical science. Nanotechnology holds the promise of democratizing healthcare by making diagnostics and treatment more accessible and cost-efficient. Nanomedicine, with its expanding applications across clinical fields such as minimally invasive surgery, chemotherapy, and organ regeneration, holds significant potential to reduce healthcare costs while enhancing patient outcomes. As regenerative technologies advance, affordable artificial tissues and organs such as skin grafts and bone implants are gradually entering the clinical landscape.

## Figures and Tables

**Table 1:** Antimicrobial effect of different nanoparticles on organisms.

Nanoparticle	Organism	Size	Ref.
Capsaicin-coated CoFe_2_O_4_ NPs	Gram-positive (*S. aureus* ATCC 52923) and Gram-negative (*E. coli* ATCC 52922)	28.1 to 31.9 nm	[62]
ZnF-CA-LP NCs	Gram-positive *Staphylococcus aureus* (*S. aureus* ATCC 25923) and Gram-negative *Escherichia coli* (*E. coli* ATCC 25922)	35 nm	[61]
ZnO NPs	*Escherichia coli*	12 to 25 nm	[63]
Au NPs	*Streptococcus pneumoniae*	134.8 nm	[64]
Ag NPs	Methicillin-resistant *Staphylococcus aureus*	20 nm	[65]
Chitosan NPs	*Staphylococcus aureus*	20.3 ± 3.2 nm	[66]
TcAg NPs	*Pseudomonas aeruginosa*, *Klebsiella pneumoniae*, *Escherichia coli*, and *Staphylococcus aureus*	125 nm	[67]

**Table 2:** Drug delivery systems: modern therapeutic agents for the oncology field.

Nanoparticle	Drug	Type of Cancer	Ref.
Chitosan–alginate NP	Alectinib	Non-small cell lung cancer	[105]
Silica-containing antioxidant NP	Sorafenib	Lung carcinoma	[106]
PEGylated liposomes	Doxorubicin and hydralazine	Breast cancer	[107]
Chitosan–albumin nanogel	Doxorubicin	Skin cancer	[108]
Zein NP	Metformin	Ehrlich carcinoma	[109]
Niosomes	Oxaliplatin or Paclitaxel	Colorectal cancer	[110]
Niosome	G-Chitosan Polymeric Platform	Lung cancer	[111]

## References

[1] Hassan A.A., Ali M.E.M., Abdel-Latif S.A., Hasani I.W., Fahim Y.A. (2025). Efficient removal of Remazol Red dye from aqueous solution using magnetic nickel ferrite nanoparticles synthesized via aqueous reflux. Sci. Rep..

[2] Hassan A.A., Fahim Y.A., Ali M.E.M. (2025). Efficient removal of Cr (VI) and As (V) from aqueous solution using magnetically separable nickel ferrite nanoparticles. J. Clust. Sci..

[3] Toumey C. (2008). Reading Feynman Into Nanotechnology: A Text for a New Science. Techne Res. Philos. Technol..

[4] Malik S., Muhammad K., Waheed Y. (2023). Nanotechnology: A revolution in modern industry. Molecules.

[5] Pautler M., Brenner S. (2010). Nanomedicine: Promises and challenges for the future of public health. Int. J. Nanomed..

[6] Conte R., Foggia R., Valentino A., Di Salle A., Kandsi F., Calarco A. (2024). Nanotechnology advancements transforming molecular diagnostics: Applications in precision healthcare. Int. J. Nano Dimens..

[7] Wong I.Y., Bhatia S.N., Toner M. (2013). Nanotechnology: Emerging tools for biology and medicine. Genes Dev..

[8] Fahim Y.A., Ragab W.M., Hasani I.W., El-Khawaga A.M. (2025). Biomedical and environmental applications via nanobiocatalysts and enzyme immobilization. Eur. J. Med. Res..

[9] Rokunuzzaman M.K. (2024). The Nanotech Revolution: Advancements in Materials and Medical Science. J. Adv. Mater. Eng..

[10] Patra J.K., Das G., Fraceto L.F., Campos E.V.R., Rodriguez-Torres M.d.P., Acosta-Torres L.S., Diaz-Torres L.A., Grillo R., Swamy M.K., Sharma S. (2018). Nano based drug delivery systems: Recent developments and future prospects. J. Nanobiotechnol..

[11] Ferraris C., Cavalli R., Panciani P.P., Battaglia L. (2020). Overcoming the blood–brain barrier: Successes and challenges in developing nanoparticle-mediated drug delivery systems for the treatment of brain tumours. Int. J. Nanomed..

[12] Mn Iqbal H., Mv Rodriguez A., Khandia R., Munjal A., Dhama K. (2017). Recent trends in nanotechnology-based drugs and formulations for targeted therapeutic delivery. Recent Pat. Inflamm. Allergy Drug Discov..

[13] Yadav A., Rathore R., Suhag D., Thakur P., Thakur A. (2025). Nanobiology of Infectious Diseases. Advancements in Nanobiology.

[14] Suhag D., Thakur P., Thakur A. (2025). Future Perspectives of Nanobiology. Advancements in Nanobiology.

[15] Shivakumar N. (2024). Recent Advances in Biological Nanodevices and Biosensors: Insights into Applications and Technological Innovations. Malays. NANO Int. J..

[16] Mabrouk M., Das D.B., Salem Z.A., Beherei H.H. (2021). Nanomaterials for biomedical applications: Production, characterisations, recent trends and difficulties. Molecules.

[17] Chen H., Zhen Z., Todd T., Chu P.K., Xie J. (2013). Nanoparticles for improving cancer diagnosis. Mater. Sci. Eng. R Rep..

[18] Mirza Z., Karim S. (2021). Nanoparticles-Based Drug Delivery and Gene Therapy for Breast Cancer: Recent Advancements and Future Challenges.

[19] Fahim Y.A., Hasani I.W., El-Khawaga A.M., Abdelhakim H.K., Sharaf N.E., Lasheen N.N. (2024). Occupational Exposure to Heavy Metal Dust and Its Hazardous Effects on Non-ferrous Foundry Workers’ Health. J. Chem. Health Risks.

[20] Koo O.M., Rubinstein I., Onyuksel H. (2005). Role of nanotechnology in targeted drug delivery and imaging: A concise review. Nanomed. Nanotechnol. Biol. Med..

[21] Batool A., Menaa F., Uzair B., Khan B.A., Menaa B. (2020). Progress and prospects in translating nanobiotechnology in medical theranostics. Curr. Nanosci..

[22] Wang J., Wang P., Shao Y., He D. (2023). Advancing treatment strategies: A comprehensive review of drug delivery innovations for chronic inflammatory respiratory diseases. Pharmaceutics.

[23] Dai X.J., Li W.J., Xie D.D., Liu B.x., Gong L., Han H.H. (2025). Stimuli-Responsive Nano Drug Delivery Systems for the Treatment of Neurological Diseases. Small.

[24] Sarella P.N.K., Vipparthi A.K., Valluri S., Vegi S., Vendi V.K. (2024). Nanorobotics: Pioneering drug delivery and development in pharmaceuticals. Res. J. Pharm. Dos. FORMS Technol..

[25] Rajendran S., Sundararajan P., Awasthi A., Rajendran S. (2023). Nanorobotics in Medicine: A Systematic Review of Advances, Challenges, and Future Prospects. arXiv.

[26] Chang Z., Fu Q., Wang M., Duan D. (2025). Advances of Nanozyme-Driven Multimodal Sensing Strategies in Point-of-Care Testing. Biosensors.

[27] Pant A., Mackraj I., Govender T. (2021). Advances in sepsis diagnosis and management: A paradigm shift towards nanotechnology. J. Biomed. Sci..

[28] Babu A., Templeton A.K., Munshi A., Ramesh R. (2014). Nanodrug delivery systems: A promising technology for detection, diagnosis, and treatment of cancer. Aaps Pharmscitech.

[29] Akhtar Z.B., Gupta A.D. (2024). Advancements within molecular engineering for regenerative medicine and biomedical applications an investigation analysis towards a computing retrospective. J. Electron. Electromed. Eng. Med. Inform..

[30] Sayed N., Allawadhi P., Khurana A., Singh V., Navik U., Pasumarthi S.K., Khurana I., Banothu A.K., Weiskirchen R., Bharani K.K. (2022). Gene therapy: Comprehensive overview and therapeutic applications. Life Sci..

[31] Mukherjee S., Suleman S., Pilloton R., Narang J., Rani K. (2022). State of the art in smart portable, wearable, ingestible and implantable devices for health status monitoring and disease management. Sensors.

[32] Sun L., Liu H., Ye Y., Lei Y., Islam R., Tan S., Tong R., Miao Y.-B., Cai L. (2023). Smart nanoparticles for cancer therapy. Signal Transduct. Target. Ther..

[33] Stojanov S., Berlec A. (2024). Smart bionanomaterials for treatment and diagnosis of inflammatory bowel disease. Nanotechnol. Rev..

[34] Murahari M.S., Yergeri M.C. (2013). Identification and usage of fluorescent probes as nanoparticle contrast agents in detecting cancer. Curr. Pharm. Des..

[35] Kumar R., Pulikanti G.R., Shankar K.R., Rambabu D., Mangili V., Kumbam L.R., Sagara P.S., Nakka N., Yogesh M. (2022). Surface coating and functionalization of metal and metal oxide nanoparticles for biomedical applications. Metal Oxides for Biomedical and Biosensor Applications.

[36] Roy D.B., Das S. (2025). Advanced Nanostructured Materials for Energy Storage Devices. Design, Fabrication, and Significance of Advanced Nanostructured Materials.

[37] Wang B., Hu S., Teng Y., Chen J., Wang H., Xu Y., Wang K., Xu J., Cheng Y., Gao X. (2024). Current advance of nanotechnology in diagnosis and treatment for malignant tumors. Signal Transduct. Target. Ther..

[38] Wani S.U.D., Ali M., Masoodi M.H., Khan N.A., Zargar M.I., Hassan R., Mir S.A., Gautam S.P., Gangadharappa H.V., Osmani R.A.M. (2022). A review on nanoparticles categorization, characterization and applications in drug delivery systems. Vib. Spectrosc..

[39] Gelperina S., Kisich K., Iseman M.D., Heifets L. (2005). The potential advantages of nanoparticle drug delivery systems in chemotherapy of tuberculosis. Am. J. Respir. Crit. Care Med..

[40] Hheidari A., Mohammadi J., Ghodousi M., Mahmoodi M., Ebrahimi S., Pishbin E., Rahdar A. (2024). Metal-based nanoparticle in cancer treatment: Lessons learned and challenges. Front. Bioeng. Biotechnol..

[41] Eleraky M.I., Razek T.M.A., Hasani I.W., Fahim Y.A. (2025). Adsorptive removal of lead, copper, and nickel using natural and activated Egyptian calcium bentonite clay. Sci. Rep..

[42] Idrees H., Zaidi S.Z.J., Sabir A., Khan R.U., Zhang X., Hassan S.-u. (2020). A review of biodegradable natural polymer-based nanoparticles for drug delivery applications. Nanomaterials.

[43] Jiao M., Zhang P., Meng J., Li Y., Liu C., Luo X., Gao M. (2018). Recent advancements in biocompatible inorganic nanoparticles towards biomedical applications. Biomater. Sci..

[44] Ayanda O.S., Mmuoegbulam A.O., Okezie O., Durumin Iya N.I., Mohammed S.a.E., James P.H., Muhammad A.B., Unimke A.A., Alim S.A., Yahaya S.M. (2024). Recent progress in carbon-based nanomaterials: Critical review. J. Nanoparticle Res..

[45] Eker F., Duman H., Akdaşçi E., Bolat E., Sarıtaş S., Karav S., Witkowska A.M. (2024). A comprehensive review of nanoparticles: From classification to application and toxicity. Molecules.

[46] Karnwal A., Jassim A.Y., Mohammed A.A., Sharma V., Al-Tawaha A.R.M.S., Sivanesan I. (2024). Nanotechnology for Healthcare: Plant-Derived Nanoparticles in Disease Treatment and Regenerative Medicine. Pharmaceuticals.

[47] Zhang L., Webster T.J. (2009). Nanotechnology and nanomaterials: Promises for improved tissue regeneration. Nano Today.

[48] Padmanabhan P., Kumar A., Kumar S., Chaudhary R.K., Gulyás B. (2016). Nanoparticles in practice for molecular-imaging applications: An overview. Acta Biomater..

[49] Barua S., Mitragotri S. (2014). Challenges associated with penetration of nanoparticles across cell and tissue barriers: A review of current status and future prospects. Nano Today.

[50] Mubeen B., Ansar A.N., Rasool R., Ullah I., Imam S.S., Alshehri S., Ghoneim M.M., Alzarea S.I., Nadeem M.S., Kazmi I. (2021). Nanotechnology as a novel approach in combating microbes providing an alternative to antibiotics. Antibiotics.

[51] Paladini F., Pollini M. (2019). Antimicrobial silver nanoparticles for wound healing application: Progress and future trends. Materials.

[52] Thwala L.N., Ndlovu S.C., Mpofu K.T., Lugongolo M.Y., Mthunzi-Kufa P. (2023). Nanotechnology-based diagnostics for diseases prevalent in developing countries: Current advances in point-of-care tests. Nanomaterials.

[53] You C., Li Q., Wang X., Wu P., Ho J.K., Jin R., Zhang L., Shao H., Han C. (2017). Silver nanoparticle loaded collagen/chitosan scaffolds promote wound healing via regulating fibroblast migration and macrophage activation. Sci. Rep..

[54] Erkoc P., Ulucan-Karnak F. (2021). Nanotechnology-based antimicrobial and antiviral surface coating strategies. Prosthesis.

[55] El-Gendy A.O., Obaid Y., Ahmed E., Enwemeka C.S., Hassan M., Mohamed T. (2022). The antimicrobial effect of gold quantum dots and femtosecond laser irradiation on the growth kinetics of common infectious eye pathogens: An in vitro study. Nanomaterials.

[56] Markandan K., Tiong Y.W., Sankaran R., Subramanian S., Markandan U.D., Chaudhary V., Numan A., Khalid M., Walvekar R. (2024). Emergence of infectious diseases and role of advanced nanomaterials in point-of-care diagnostics: A review. Biotechnol. Genet. Eng. Rev..

[57] Makabenta J.M.V., Nabawy A., Li C.-H., Schmidt-Malan S., Patel R., Rotello V.M. (2021). Nanomaterial-based therapeutics for antibiotic-resistant bacterial infections. Nat. Rev. Microbiol..

[58] Tang Y., Xu H., Wang X., Dong S., Guo L., Zhang S., Yang X., Liu C., Jiang X., Kan M. (2023). Advances in preparation and application of antibacterial hydrogels. J. Nanobiotechnol..

[59] Hamdan N., Yamin A., Hamid S.A., Khodir W.K.W.A., Guarino V. (2021). Functionalized antimicrobial nanofibers: Design criteria and recent advances. J. Funct. Biomater..

[60] Ifijen I.H., Omonmhenle S.I. (2024). Antimicrobial properties of carbon nanotube: A succinct assessment. Biomed. Mater. Devices.

[61] Fahim Y.A., El-Khawaga A.M., Sallam R.M., Elsayed M.A., Assar M.F.A. (2024). Immobilized lipase enzyme on green synthesized magnetic nanoparticles using Psidium guava leaves for dye degradation and antimicrobial activities. Sci. Rep..

[62] El-Khawaga A.M., Elsayed M.A., Fahim Y.A., Shalaby R.E. (2023). Promising photocatalytic and antimicrobial activity of novel capsaicin coated cobalt ferrite nanocatalyst. Sci. Rep..

[63] Elabbasy M.T., El Bayomi R.M., Abdelkarim E.A., Hafez A.E.-S.E., Othman M.S., Ghoniem M.E., Samak M.A., Alshammari M.H., Almarshadi F.A., Elsamahy T. (2025). Antibacterial and Antibiofilm Activity of Green-Synthesized Zinc Oxide Nanoparticles Against Multidrug-Resistant Escherichia coli Isolated from Retail Fish. Molecules.

[64] Azmy L., Al-Olayan E., Abdelhamid M.A.A., Zayed A., Gheda S.F., Youssif K.A., Abou-Zied H.A., Abdelmohsen U.R., Ibraheem I.B.M., Pack S.P. (2024). Antimicrobial Activity of Arthrospira Platensis-Mediated Gold Nanoparticles against Streptococcus Pneumoniae: A Metabolomic and Docking Study. Int. J. Mol. Sci..

[65] Fareid M.A., El-Sherbiny G.M., Askar A.A., Abdelaziz A.M., Hegazy A.M., Ab Aziz R., Hamada F.A. (2025). Impeding Biofilm-Forming Mediated Methicillin-Resistant Staphylococcus aureus and Virulence Genes Using a Biosynthesized Silver Nanoparticles–Antibiotic Combination. Biomolecules.

[66] Godoy C.A., Balic I., Moreno A.A., Diaz O., Arenas Colarte C., Bruna Larenas T., Gamboa A., Caro Fuentes N. (2025). Antimicrobial and Antibiofilm Activity of Chitosan Nanoparticles Against Staphylococcus aureus Strains Isolated from Bovine Mastitis Milk. Pharmaceutics.

[67] Lekkala V.D.V.V., Muktinutalapati A.V., Lebaka V.R., Lomada D., Korivi M., Li W., Reddy M.C. (2025). Green Synthesis and Characterization of Silver Nanoparticles from Tinospora cordifolia Leaf Extract: Evaluation of Their Antioxidant, Anti-Inflammatory, Antibacterial, and Antibiofilm Efficacies. Nanomaterials.

[68] Alshammari T.M. (2016). Drug safety: The concept, inception and its importance in patients’ health. Saudi Pharm. J..

[69] Fahim Y.A., Hasani I.W., Mahmoud Ragab W. (2025). Promising biomedical applications using superparamagnetic nanoparticles. Eur. J. Med. Res..

[70] Singh R., Kumar S. (2022). Cancer targeting and diagnosis: Recent trends with carbon nanotubes. Nanomaterials.

[71] Ruiz-Hitzky E., Darder M., Aranda P., Ariga K. (2010). Advances in biomimetic and nanostructured biohybrid materials. Adv. Mater..

[72] Buonanno A., Salvatore M.M., Feola A., Siciliano A., Bellavita R., Imbò L.E., Guida M., Andolfi A., Nicoletti R., Maione A. (2024). Sphaeropsidin A Loaded in Liposomes to Reduce Its Cytotoxicity and Preserve Antifungal Activity Against *Candida auris*. Molecules.

[73] Asadullah Khan M., Peng R., Liu C., Chen Z. (2022). Synthesis, dynamics and applications (cytotoxicity and biocompatibility) of dendrimers: A mini-review. Eur. Polym. J..

[74] Sikora J., Błaszkiewicz P., Dudkowiak A., Jagielska J., Żurawski J. (2024). Cytotoxicity of gold nanoparticles to human lymphocytes: A comparison between rod-shaped and spherical nanoparticles. Contemp. Oncol./Współczesna Onkol..

[75] Yadav K. (2024). Nanotechnology in diabetes management: Revolutionizing treatment and diagnostics. J. Mol. Liq..

[76] Singh V. (2024). Theranostics: Integrated Diagnostics and Therapy Using Nanomedicine. Nanomedicine.

[77] Savaliya R., Shah D., Singh R., Kumar A., Shanker R., Dhawan A., Singh S. (2015). Nanotechnology in disease diagnostic techniques. Curr. Drug Metab..

[78] Ahmed M.U., Saaem I., Wu P.C., Brown A.S. (2014). Personalized diagnostics and biosensors: A review of the biology and technology needed for personalized medicine. Crit. Rev. Biotechnol..

[79] Woźniak M., Płoska A., Siekierzycka A., Dobrucki L.W., Kalinowski L., Dobrucki I.T. (2022). Molecular imaging and nanotechnology—Emerging tools in diagnostics and therapy. Int. J. Mol. Sci..

[80] Fuchigami T., Nakayama T., Miyanari Y., Nozaki I., Ishikawa N., Tagawa A., Yoshida S., Munekane M., Nakayama M., Ogawa K. (2024). Peptide-Based Turn-On Fluorescent Probes for Highly Specific Detection of Survivin Protein in the Cancer Cells. Chem. Biomed. Imaging.

[81] Minges P., Eder M., Eder A.-C. (2025). Dual-Labeled Small Peptides in Cancer Imaging and Fluorescence-Guided Surgery: Progress and Future Perspectives. Pharmaceuticals.

[82] Rafiq Z., Patel P., Kumar S., Sofi H.S., Macossay J., Sheikh F.A. (2020). Advancements of Nanotechnology in Diagnostic Applications. Application of Nanotechnology in Biomedical Sciences.

[83] Gallo E., Diaferia C., Balasco N., Sibillano T., Roviello V., Giannini C., Vitagliano L., Morelli G., Accardo A. (2021). Fabrication of fluorescent nanospheres by heating PEGylated tetratyrosine nanofibers. Sci. Rep..

[84] Li P., Lee G.-H., Kim S.Y., Kwon S.Y., Kim H.-R., Park S. (2021). From diagnosis to treatment: Recent advances in patient-friendly biosensors and implantable devices. ACS Nano.

[85] Huang Q., Wang Y., Chen X., Wang Y., Li Z., Du S., Wang L., Chen S. (2018). Nanotechnology-based strategies for early cancer diagnosis using circulating tumor cells as a liquid biopsy. Nanotheranostics.

[86] Taha B.A., Kadhim A.C., Addie A.J., Haider A.J., Azzahrani A.S., Raizada P., Rustagi S., Chaudhary V., Arsad N. (2024). Advancing cancer diagnostics through multifaceted optical biosensors supported by nanomaterials and artificial intelligence: A panoramic outlook. Microchem. J..

[87] Manzetti S., Vasilache D., Francesco E. (2015). Emerging carbon-based nanosensor devices: Structures, functions and applications. Adv. Manuf..

[88] Wanas W., Khalifa D.H., Gamal H., El-Kot S.M. (2024). Nanotechnology for Cancer Research (Diagnosis and Therapy): Recent Progress and Future Prospects. Interdisciplinary Cancer Research.

[89] Sengar A. (2024). Precision in practice: Nanotechnology and targeted therapies for personalized care. Int. J. Adv. Nano Comput. Anal..

[90] Haleem A., Javaid M., Singh R.P., Suman R., Rab S. (2021). Biosensors applications in medical field: A brief review. Sens. Int..

[91] Shen Z., Wu A., Chen X. (2017). Iron oxide nanoparticle based contrast agents for magnetic resonance imaging. Mol. Pharm..

[92] Nyström A.M., Fadeel B. (2012). Safety assessment of nanomaterials: Implications for nanomedicine. J. Control. Release.

[93] Walmsley G.G., McArdle A., Tevlin R., Momeni A., Atashroo D., Hu M.S., Feroze A.H., Wong V.W., Lorenz P.H., Longaker M.T. (2015). Nanotechnology in bone tissue engineering. Nanomed. Nanotechnol. Biol. Med..

[94] Nethi S.K., Das S., Patra C.R., Mukherjee S. (2019). Recent advances in inorganic nanomaterials for wound-healing applications. Biomater. Sci..

[95] Malik S., Sundarrajan S., Hussain T., Nazir A., Ayyoob M., Berto F., Ramakrishna S. (2021). Sustainable nanofibers in tissue engineering and biomedical applications. Mater. Des. Process. Commun..

[96] Huang J., Liu F., Su H., Xiong J., Yang L., Xia J., Liang Y. (2022). Advanced nanocomposite hydrogels for cartilage tissue engineering. Gels.

[97] Singh S. (2010). Nanomedicine–nanoscale drugs and delivery systems. J. Nanosci. Nanotechnol..

[98] Muteeb G., El-Morsy M.T., Abo-Taleb M.A., Mohamed S.K., Khafaga D.S.R. (2025). Herbal Medicine: Enhancing the Anticancer Potential of Natural Products in Hepatocellular Carcinoma Therapy Through Advanced Drug Delivery Systems. Pharmaceutics.

[99] Khafaga D.S.R., Muteeb G., Aswa D.W., Aatif M., Farhan M., Allam S. (2025). Green chemistry: Modern therapies using nanocarriers for treating rare brain cancer metastasis from colon cancer. SLAS Discov..

[100] Muteeb G., Khafaga D.S.R., El-Morsy M.T., Farhan M., Aatif M., Hosney M. (2024). Targeting tumor-associated macrophages with nanocarrier-based treatment for breast cancer: A step toward developing innovative anti-cancer therapeutics. Heliyon.

[101] Perinelli D.R., Cespi M., Bonacucina G., Palmieri G.F. (2019). PEGylated polylactide (PLA) and poly (lactic-co-glycolic acid)(PLGA) copolymers for the design of drug delivery systems. J. Pharm. Investig..

[102] Kalasin S., Surareungchai W. (2023). Challenges of emerging wearable sensors for remote monitoring toward telemedicine healthcare. Anal. Chem..

[103] Egwu C.O., Aloke C., Onwe K.T., Umoke C.I., Nwafor J., Eyo R.A., Chukwu J.A., Ufebe G.O., Ladokun J., Audu D.T. (2024). Nanomaterials in drug delivery: Strengths and opportunities in medicine. Molecules.

[104] Herdiana Y., Wathoni N., Gozali D., Shamsuddin S., Muchtaridi M. (2023). Chitosan-based nano-smart drug delivery system in breast cancer therapy. Pharmaceutics.

[105] Ata T.e., Al-Ani I., Karameh N., Atta M.R., Dayyih W.A. (2025). Alectinib-Loaded Chitosan–Alginate Nanoparticles: A Novel Synthesis Method with In Vitro and In Vivo Evaluations. Pharmaceutics.

[106] Shashni B., Tran H.T., Vong L.B., Chung R.-J., Nagasaki Y. (2025). Sorafenib-Loaded Silica-Containing Redox Nanoparticle Decreases Tumorigenic Potential of Lewis Lung Carcinoma. Pharmaceutics.

[107] Alshaer W., Lafi Z., Nsairat H., AlQuaissi B., Alqudah D.A., Zureigat H., Hamad I. (2025). Remote Co-Loading of Doxorubicin and Hydralazine into PEGylated Liposomes: In Vitro Anti-Proliferative Effect Against Breast Cancer. Molecules.

[108] Radeva L., Zaharieva M.M., Spassova I., Kovacheva D., Pencheva-El Tibi I., Najdenski H., Yoncheva K. (2024). Biopolymeric Nanogel as a Drug Delivery System for Doxorubicin—Improved Drug Stability and Enhanced Antineoplastic Activity in Skin Cancer Cells. Pharmaceuticals.

[109] Elmahboub Y., Albash R., Magdy William M., Rayan A.H., Hamed N.O., Ousman M.S., Raslan N.A., Mosallam S. (2024). Metformin loaded zein polymeric nanoparticles to augment antitumor activity against Ehrlich carcinoma via activation of AMPK pathway: D-optimal design optimization, in vitro characterization, and in vivo study. Molecules.

[110] El-Far S.W., Abo El-Enin H.A., Abdou E.M., Nafea O.E., Abdelmonem R. (2022). Targeting colorectal cancer cells with niosomes systems loaded with two anticancer drugs models; comparative in vitro and anticancer studies. Pharmaceuticals.

[111] Zarepour A., Egil A.C., Cokol Cakmak M., Esmaeili Rad M., Cetin Y., Aydinlik S., Ozaydin Ince G., Zarrabi A. (2023). Fabrication of a dual-drug-loaded smart niosome-g-chitosan polymeric platform for lung cancer treatment. Polymers.

[112] Weerarathna I.N., Kumar P., Dzoagbe H.Y., Kiwanuka L. (2025). Advancements in Micro/Nanorobots in Medicine: Design, Actuation, and Transformative Application. ACS Omega.

[113] Kong X., Gao P., Wang J., Fang Y., Hwang K.C. (2023). Advances of medical nanorobots for future cancer treatments. J. Hematol. Oncol..

[114] Kavousinejad S. (2024). Simulation of Nanorobots with Artificial Intelligence and Reinforcement Learning for Advanced Cancer Cell Detection and Tracking. arXiv.

[115] Li J., Esteban-Fernández de Ávila B., Gao W., Zhang L., Wang J. (2017). Micro/nanorobots for biomedicine: Delivery, surgery, sensing, and detoxification. Sci. Robot..

[116] Soto F., Wang J., Ahmed R., Demirci U. (2020). Medical micro/nanorobots in precision medicine. Adv. Sci..

[117] Jeyachandran S., Srinivasan R., Ramesh T., Parivallal A., Lee J., Sathiyamoorthi E. (2023). Recent development and application of “nanozyme” artificial enzymes—A review. Biomimetics.

[118] Shi X., Liu J., Wang G. (2023). A peroxidase-like magneto-gold nanozyme AuNC@ Fe_3_O_4_ with photothermal effect for induced cell apoptosis of hepatocellular carcinoma cells in vitro. Front. Bioeng. Biotechnol..

[119] Jiang B., Yan L., Zhang J., Zhou M., Shi G., Tian X., Fan K., Hao C., Yan X. (2019). Biomineralization synthesis of the cobalt nanozyme in SP94-ferritin nanocages for prognostic diagnosis of hepatocellular carcinoma. ACS Appl. Mater. Interfaces.

[120] Sen A., Oswalia J., Yadav S., Vachher M., Nigam A. (2024). Recent trends in nanozyme research and their potential therapeutic applications. Curr. Res. Biotechnol..

[121] Kwatra D., Venugopal A., Anant S. (2013). Nanoparticles in radiation therapy: A summary of various approaches to enhance radiosensitization in cancer. Transl. Cancer Res..

[122] Arif M., Nawaz A.F., Ullah Khan S., Mueen H., Rashid F., Hemeg H.A., Rauf A. (2023). Nanotechnology-based radiation therapy to cure cancer and the challenges in its clinical applications. Heliyon.

[123] Mazumdar H., Khondakar K.R., Das S., Halder A., Kaushik A. (2025). Artificial intelligence for personalized nanomedicine; from material selection to patient outcomes. Expert Opin. Drug Deliv..

[124] Shapira A., Livney Y.D., Broxterman H.J., Assaraf Y.G. (2011). Nanomedicine for targeted cancer therapy: Towards the overcoming of drug resistance. Drug Resist. Updates.

[125] Chandrakala V., Aruna V., Angajala G. (2022). Review on metal nanoparticles as nanocarriers: Current challenges and perspectives in drug delivery systems. Emergent Mater..

[126] Salvador-Morales C., Grodzinski P. (2022). Nanotechnology tools enabling biological discovery. ACS Nano.

[127] Lenar N., Paczosa-Bator B. (2025). Nanosensors and Microsensors for Body Fluid Monitoring: Various Analyte Detection and Construction Solutions. Int. J. Mol. Sci..

